# Exhaled breath analysis: a review of ‘breath-taking’ methods for off-line analysis

**DOI:** 10.1007/s11306-017-1241-8

**Published:** 2017-08-19

**Authors:** Oluwasola Lawal, Waqar M. Ahmed, Tamara M. E. Nijsen, Royston Goodacre, Stephen J. Fowler

**Affiliations:** 1 Division of Infection, Immunity and Respiratory Medicine, School of Biological Sciences, Faculty of Biology, Medicine and Health, The University of Manchester, Manchester, UK; 2 Philips Research, Royal Philips B.V., Eindhoven, The Netherlands; 3 School of Chemistry, Manchester Institute of Biotechnology, The University of Manchester, Manchester, UK; 4 Manchester Academic Health Science Centre, The University of Manchester and University Hospital of South Manchester NHS Foundation Trust, Manchester, UK

**Keywords:** Breath sampling, Breath phases, Breath collection, Breath pre-concentration

## Abstract

**Background:**

The potential of exhaled breath sampling and analysis has long attracted interest in the areas of medical diagnosis and disease monitoring. This interest is attributed to its non-invasive nature, access to an unlimited sample supply (i.e., breath), and the potential to facilitate a rapid at patient diagnosis. However, progress from laboratory setting to routine clinical practice has been slow. Different methodologies of breath sampling, and the consequent difficulty in comparing and combining data, are considered to be a major contributor to this. To fulfil the potential of breath analysis within clinical and pre-clinical medicine, standardisation of some approaches to breath sampling and analysis will be beneficial.

**Objectives:**

The aim of this review is to investigate the heterogeneity of breath sampling methods by performing an in depth bibliometric search to identify the current state of art in the area. In addition, the review will discuss and critique various breath sampling methods for off-line breath analysis.

**Methods:**

Literature search was carried out in databases MEDLINE, BIOSIS, EMBASE, INSPEC, COMPENDEX, PQSCITECH, and SCISEARCH using the STN platform which delivers peer-reviewed articles. Keywords searched for include breath, sampling, collection, pre-concentration, volatile. Forward and reverse search was then performed on initially included articles. The breath collection methodologies of all included articles was subsequently reviewed.

**Results:**

Sampling methods differs between research groups, for example regarding the portion of breath being targeted. Definition of late expiratory breath varies between studies.

**Conclusions:**

Breath analysis is an interdisciplinary field of study using clinical, analytical chemistry, data processing, and metabolomics expertise. A move towards standardisation in breath sampling is currently being promoted within the breath research community with a view to harmonising analysis and thereby increasing robustness and inter-laboratory comparisons.

**Electronic supplementary material:**

The online version of this article (doi:10.1007/s11306-017-1241-8) contains supplementary material, which is available to authorized users.

## Introduction

Breath odours were used for disease recognition long before present-day diagnostics; a sweet smell was associated with diabetes mellitus, fish-like smell with liver disease, and urine-like smell with kidney disease (Phillips 1992). Exhaled breath is predominantly composed of nitrogen, oxygen, carbon dioxide, argon as well as water vapour, whereas the volatile organic compounds (VOCs) which may be diagnostically useful are only found in trace concentrations (Lourenco and Turner 2014). Identification of VOCs patterns via human olfaction for disease diagnosis is of course subjective, and modern analytical instruments have sought to make this more reliable and robust. This has led to a sustained interest in breath diagnosis due to its non-invasive nature, with the ability to take repeat measurements with little stress or discomfort to the individual under investigation.

The concept behind breath metabolomics (also known as breathomics) is that the VOC profile in breath will be altered when a switch from a healthy to a pathological state occurs and this can be detected and potentially utilised for diagnosis and monitoring (Beale et al. 2016). The origin of breath VOCs include the environment (termed exogenous), the host (endogenous), and also the microbiome (the microorganisms that inhabit the mouth, lung and gut) (Boots et al. 2015; Bos et al. 2013; Schulz and Dickschat 2007). Most of the identified VOCs in exhaled breath originate exogenously (Costello et al. 2014), but endogenous and microbial VOCs are of more interest clinically. Endogenous VOCs have the potential to provide a snapshot of the physiological state of an individual whilst microbial VOCs could aid in pathogen identification, and the interaction of the host and commensal microorganisms contributes significant complexity to the metabolome of this complex “superorganism” (Goodacre 2007). Breath VOCs are found at trace levels [typically parts per million volume (ppmv) and lower] and their reliable detection poses a challenge, hence the typical use of sample pre-concentration coupled with a highly sensitive analytical instrument. Several methods are utilised for pre-concentration using thermal desorption chemistries such as solid phase microextraction (SPME) and needle trap devices (NTDs).

**Fig. 1:**
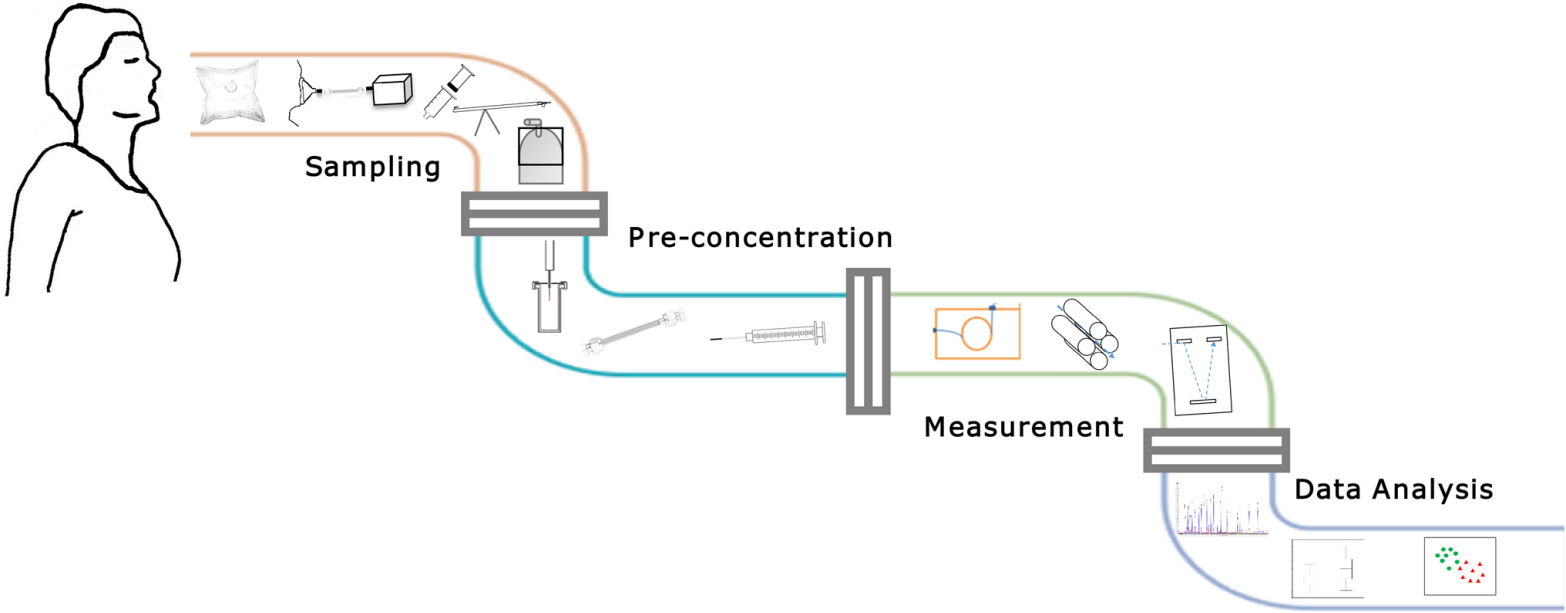
A diagram illustrating the off-line breath sampling pipeline. First section shows breath sampling containers [From L to R gas sampling bag, face-mask, Bio-VOC™ Sampler, breath collection apparatus (BCA), canister], second section indicates pre-concentration methods [From L to R solid phase microextraction (SPME), Thermal desorption (TD) tube, needle trap devices (NTDs)]. Gas chromatograph (GC) and mass analysers [quadrupole, time-of-flight (Tof)] are in the third section and the fourth section depicts targeted and untargeted data pre-treatment, processing and analysis

The off-line breathomics pipeline can be broadly broken down into breath sample collection, sample analysis, and data analysis (Rattray et al. 2014). There are several ways of achieving the desired goal in each section. For example, for breath sample collection, factors such as type of breath to be collected (i.e., mixed expiratory or end-tidal), single or multiple exhalation, and choice of breath capture technology are just some of the options to be considered (Fig. 1).

In this article, a summary of methods utilised in the off-line breathomics pipeline across studies is shown, whilst evaluating heterogeneity between methods and suggesting where standardisation may be beneficial.

## Breathomics bibliography search criteria

The search was conducted to include articles until the 24th of October 2016 and the overall process used is schematically shown in the supplementary material (Figure S1). To ensure quality, the STN platform (https://www.stn.org/stn/) was utilised which delivers peer reviewed articles only. Databases searched include MEDLINE, BIOSIS, EMBASE, INSPEC, COMPENDEX, PQSCITECH, and SCISEARCH. The search strategy included looking for (breath?) in article titles and ((sampl? OR collect? OR pre(W)concentrat? OR preconcentrat?) AND volatile) in the title and abstracts of articles. “?” and “W” denotes any number of characters to the right of the term and one character between two words (e.g. space or hyphen) respectively. In total, 395 manuscripts were obtained after automatic filtration. These articles were subsequently manually filtered to exclude review articles, breath condensate articles, articles with only real time analytical platforms, and also non-human studies. In the scenario where the same breath sampling methodology is used by the same first author in multiple articles, only one of the articles was included. To prevent bias, articles with the same breath sampling methodology and same last author (or thought to originate from the same laboratory) were classified together as one for descriptive statistics purposes. Additional articles were included following forward and reverse searching resulting in the final inclusion of 110 papers. From the final 110 articles the following parameters were assessed:

- Exhaled breath portion targeted
- Breath collection container
- Pre-concentration methods

**Fig. 2:**
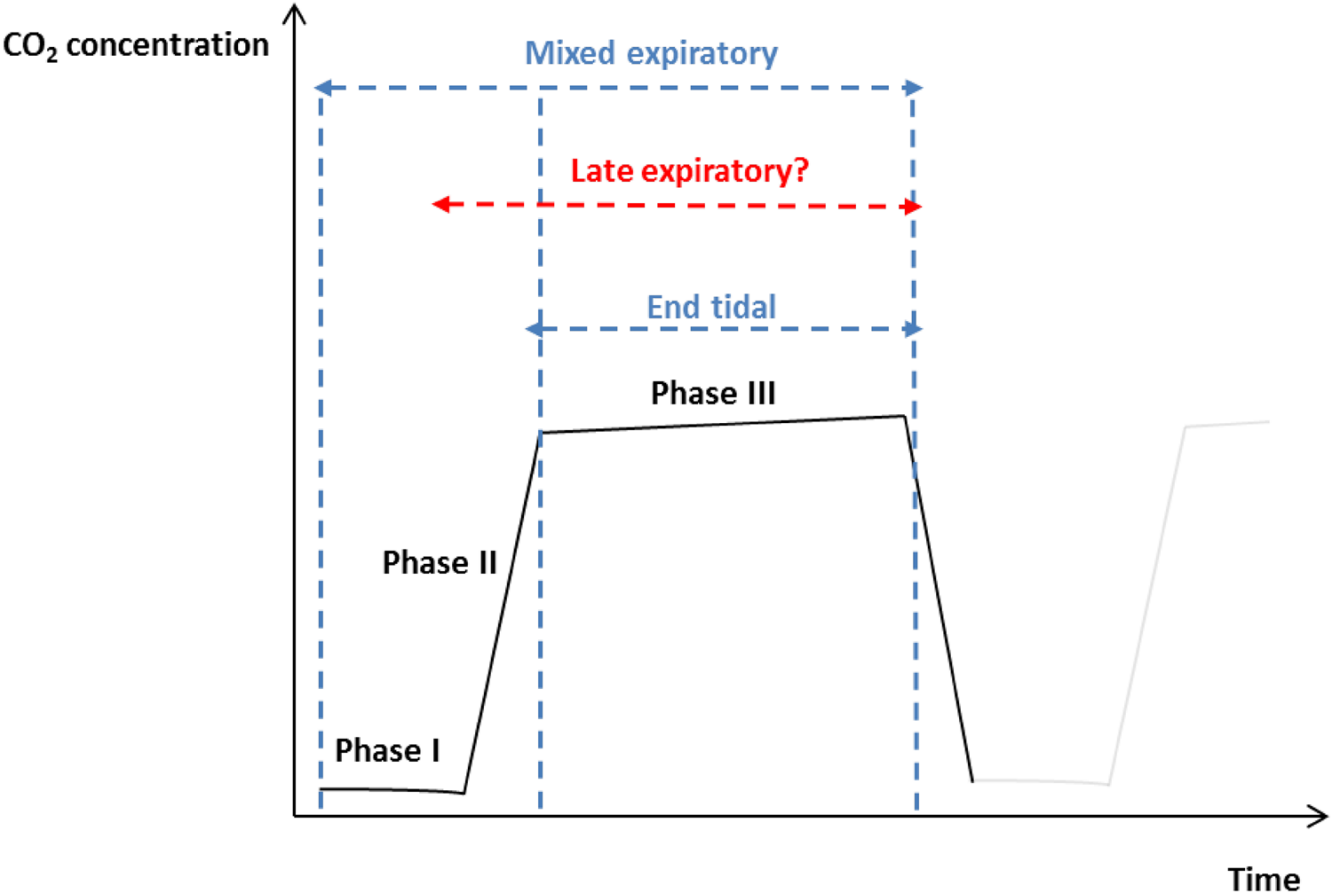
Schematic visual representation depicting a single exhaled breath phases by capnography. Late expiratory breath is undefined as there is no standard practice for collecting this breath type and definitions vary. Phase I dead space, Phase II transition, Phase III alveolar. Adapted from (Miekisch et al. 2008)

A schematic representation of exhaled breath phases is depicted in Fig. 2 and thus the breath type categories were sub-categorised into: late expiratory breath (Table 1), sampling from the end-tidal or ‘alveolar’ breath (Table 2), and mixed expiratory breath (Table 3). Other breath types can be found in the supplementary information (Table S1).These tables include a brief description of how the breath type was obtained, and further categorisation was performed according to the type of breath collection container, as well as the pre-concentration method employed in the study.

**Table 1:** Studies that collected late expiratory breath

Brief description	Breath collection container	Pre-concentration method	Reference
Dead space air collected in one bag, late expired air in separate bag	Tedlar bag	TD tube—Multi-bed ORBO™ 420/Tenax TA TD tube—Tenax	(Amal et al. 2016; Barash et al. 2015; Gruber et al. 2014; Peled et al. 2012; Xu et al. 2013)
First 10 s excluded from bag	Tedlar bag	TD tube—Carboxen 1000/Carbopack X/Carbopack B	(Sanchez and Sacks 2006)
Deep breath, held for 10 s, exhaled slowly for 10 s prior to filling bag	Tedlar bag	TD tube—Carbopack Y/B/X/Carboxen 1000	(Libardoni et al. 2006)
Exhale air into tube connected to bag	Tedlar bag	TD tube—Tenax GC	(Preti et al. 1988)
Forced expiratory breath with first 2–3 s of expiration not collected	Tedlar bag	In house sorbent microtrap—Carboxen 1000, Carbopack X, Carbopack B	(Castellanos et al. 2016)
Final portion of exhaled breath collected	ALTEF polypropylene bag	TD tube—Tenax TA	(Harshman et al. 2015)
Last portion of breath collected using ha-pause method	Aluminium gas bag	TD tube—Tenax TA/Unicarb	(Berna et al. 2015)
Second half of exhaled breath collected	FlexFilm bag	TD tube—Tenax TA/Carbotrap B	(Bigazzi et al. 2016)
First third of each breath not collected	Tedlar bag	SPME—75 µM CAR/PDMS	(Chen et al. 2016)
Dead space air collected in one bag, late expired air in separate bag Mixed expiratory	Tedlar bag	SPME—75 µM CAR/PDMS	(Capuano et al. 2015)
Dead space air collected in one bag, late expired air in separate bag	Tedlar bag	SPME—100 µM PDMS, 65 µM PDMS/DVB	(Ma et al. 2014)
Last portion of exhaled breath using breath collecting device	Tedlar bag	SPME—100 µM PDMS	(Wang et al. 2012)
Dead space air collected in one bag, late expired air in separate bag	Tedlar bag	SPME—fiber not specified	(Santonico et al. 2012)
Dead space air collected in one bag, late expired air in separate bag	Mylar bag	SPME—PDMS/DVB	(Hakim et al. 2011) (Peng et al. 2010)
Last of exhaled breath (150 mL)	Bio-VOC	TD tube—Carbotrap 300	(Das et al. 2014)
Deep breath and slowly exhale as fully as possible	Bio-VOC	TD tube—Carbograph 1TD/Carbopack X	(Phillips et al. 2014)
Retained last of exhaled breath (~ 100 mL)	Bio-VOC	TD tube—Tenax TA/Unicarb	(Zaric et al. 2014)
End of forced vital capacity collected	Bio-VOC	TD tube—Tenax TA/Graphitized Carbon black/Carbonized molecular sieve	(Jareno-Esteban et al. 2013)
Last portion of exhaled breath (~150 mL)	Bio-VOC	TD tube—Tenax TA/Unicarb	(Dadamio et al. 2012)
Last 150 mL of single slow vital capacity Mouth air	Bio-VOC	TD tube—Tenax TA/Unicarb	(van den Velde et al. 2007)
Exhale until lungs are empty	Bio-VOC	TD tube—sorbent not specified	(Henderson and Matthews 2002)
Blow deeply and slowly through sampler (150 mL)	Bio-VOC	SPME—75 µM CAR/PDMS	(Raninen et al. 2016)
Last 150 mL of single slow vital capacity	Bio-VOC	SPME—75 µM CAR/PDMS, 65 µM PDMS/DVB	(Corradi et al. 2015)
Last portion of exhaled breath (~100 mL)	Bio-VOC	SPME—75 µM CAR/PDMS	(Kramer et al. 2015)
Last 150 mL of single slow vital capacity	Bio-VOC	SPME—75 µM CAR/PDMS	(Poli et al. 2008)
Exhale into tubular structure with dead space air flowing downstream and air collected upstream	BCA	TD tube—Carbotrap/Carbosieve SIII	(Zeliger et al. 2012) (Moretti et al. 2004) (Phillips 1997)
18 mL of one single end-tidal exhalation portion	Gas-tight syringe, SPME vial	SPME—75 µM CAR/PDMS	(King et al. 2010)
Single exhalations with first 750 mL discarded	Gas-tight syringe, glass vial	SPME—65 µM PDMS/DVB	(Svensson et al. 2007)
Take 2–3 deep breaths, inhale and hold breath for 10–15 s, exhale into glass tube and collect an aliquot of end- expired air	Glass tube, aluminum tube	SPME—100 µM CAR/PDMS TD tube—Tenax TA	(Prado et al. 2003)
Pressure sensors to estimate breath phases	Face mask	TD tube—Tenax TA/Carbograph 1TD	(Kang and Paul Thomas 2016) (Turner et al. 2011)
Pressure sensors to estimate breath phases	Face mask	TD tube—Tenax TA/Carbotrap	(Basanta et al. 2010) (Ibrahim et al. 2011)
First portion of breath removed	Breath device connected to desorption tube	TD tube—Carbopack B/Carbopack C	(Khalid et al. 2013)
Collected 1000 mL after discarding dead space	Glass container	TD tube—Tenax	(Mangler et al. 2012)
Two tidal volume ventilations, a deep inspiration and slow exhalation for 10 s, first 3 s discarded	Stainless steel canisters	Glass beads	(Minh et al. 2011)
Deep inspiration, 5 s breathhold, slow and complete exhalation over 10 s. First 2 s discarded	Electro-polished stainless steel	–	(Barker et al. 2006)

*BCA* breath collection apparatus, *CAR* carboxen, *DMS* differential mobility spectrometry, *DVB* divinylbenzene, *ECD* electron capture detector, *FID* flame ionisation detector, *GC-MS* gas chromatography-mass spectrometry, *PDMS* polydimethylsiloxane, *TD* thermal desorption, *Tof* time of flight

**Table 2:** Studies that collected end-tidal or ‘alveolar’ breath

Brief description	Breath collection container	Pre-concentration method	Reference
CO_2_ visual control & mixed expiratory using plastic straws	Tedlar bag	TD tube—Tenax TA/Carboxen 569/Carboxen 1000	(Filipiak et al. 2014)
CO_2_ visual control	Tedlar bag	TD tube—Tenax TA	(Grabowska-Polanowska et al. 2013)
CO_2_ visual control	Nalophan bag	TD tube—Tenax GR	(Salvo et al. 2015)
CO_2_ visual control	Tedlar bag	SPME—75 µM CAR/PDMS	(Mochalski et al. 2014)
CO_2_ visual control	Tedlar bag Gas-tight syringe, glass vial	SPME—PDMS, PDMS/DVB, PA, CAR/PDMS, CW/DVB, DVB/CAR/PDMS SPME—75 µM CAR/PDMS	(Bajtarevic et al. 2009; Buszewski et al. 2009; Ligor et al. 2007; Ulanowska et al. 2011; Ulanowska et al. 2012)
CO_2_ visual control	Tedlar bag Gas-tight syringe, glass vial	SPME—CAR/PDMS	(Miekisch et al. 2008)
CO_2_ visual control	Tedlar bag	NTD—Tenax TA/Carbopack X/Carboxen 1000	(Mochalski et al. 2013)
CO_2_ visual control	–	NTD—Tenax TA/Carbopack X/Carboxen 1000	(Gruber et al. 2016)
CO_2_ visual control	–	NTD—Tenax TA/Carbopack X/Carboxen 1000	(Mieth et al. 2010)
CO_2_ visual control	Gas-tight syringe, glass vial	SPME—75 µM CAR/PDMS	(Guo et al. 2015; Wang et al. 2014)
CO_2_ visual control	Gas-tight syringe, glass vial	SPME—CAR/PDMS SPME—75 µM CAR/PDMS SPME—65 µM PDMS/DVB	(Fuchs et al. 2010; Goerl et al. 2013; Kischkel et al. 2012; Pabst et al. 2007; Schubert et al. 2005)
Rebreathed air	Tedlar bag	Freeze-trap breath in glass U-tube	(Jones et al. 1983)
*CO_2_ visual control	glass syringe	TD tube—Carbotrap B/Carbopack X	(Filipiak et al. 2015)

*CW* carbowax, *NTD* needle trap device, asterisk (*) denotes ventilated patients

**Table 3:** Studies that collected mixed expiratory breath

Brief description	Breath collection container	Pre-concentration method	Reference
Breathe through to face mask Exhalation into bag Inhale hold breath for 5 s and fully expire	Tedlar bag	TD tube—Carbograph 1TD/Carbopack X TD tube—Carbon-filled	(Pijls et al. 2016) (Smolinska et al. 2014) (Baranska et al. 2013; Dallinga et al. 2010; Robroeks et al. 2010; Van Berkel et al. 2010; van de Kant et al. 2013; Verdam et al. 2013)
Single vital capacity following deep inspiration	Tedlar bag	TD tube—Carboxen 1003/Carbopack B/Carbopack Y	(Altomare et al. 2013)
Forced expiration	Tedlar, Supel foil, Supel inert gas sampling bags. Glass sampling bulbs	TD tube-Chromosorb106/Tenax TA/Carbopack B SPME—DVB/CAR/PDMS	(Scott-Thomas et al. 2013)
Deep breath and exhale	Tedlar bag	TD tube—Carboxen 1000/Carbopack X /Carbopack B	(Alonso et al. 2010)
Spirometer used	Tedlar bag	TD tube—Tenax	(Gordon et al. 1988)
Inhale air to total lung capacity and exhale into bag	Mylar bag	TD tube—sorbent not specified	(Machado et al. 2005)
Breathe moderately into bag after initial washout period	Tedlar bag	SPME—75 µM CAR/PDMS	(Hyspler et al. 2000)
Inhale/exhale normally then deeply exhale into bag after 5 s holding breath	Tedlar bag	SPME—50/30 µM DVB/CAR/PDMS	(Caldeira et al. 2012)
Deeply breathe into bag	Tedlar bag	SPME—75 µM CAR/PDMS	(Song et al. 2010)
Breath collected using straw	Tedlar bag	SPME—75 µM CAR/PDMS	(Bajtarevic et al. 2009; Ligor et al. 2009)
Exhale into bag via straw	Tedlar bag	SPME—CAR/PDMS	(Erhart et al. 2009)
Inhale moderately and exhale as much as possible	Tedlar bag	SPME—PDMS/DVB	(Deng et al. 2004)
Expired into a bag via a rudolph valve and delivery tube	Gas sampling bag	TD tube—Tenax GC	(Gordon et al. 1985)
Inspired/expired deeply 3×, retained breath for 20 s and then expired into container	Bio-VOC	TD tube—Tenax TA	(Marco and Grimalt 2015)
Breathe deeply through breath collection container	Bio-VOC, ALTEF polypropylene bag	TD tube—Tenax TA	(Kwak et al. 2014)
Breathe at normal frequency through RTube	RTube	SPME—65 µM PDMS/DVB	(Martin et al. 2010)
Deep inhalation and slow exhalation through sampling device	Gas bulb	SPME—75 µM CAR/PDMS	(Schallschmidt et al. 2016)
Forced expiration of five breaths	Gas bulb	SPME—DVB/CAR/PDMS	(Syhre et al. 2009)
Inhaled through a carbon filter and exhaled into a reservoir	Stainless steel reservoir	TD tube—Tenax TA	(Gaida et al. 2016)
Whole breath sample collected	Stainless steel canister	–	(Gordon et al. 2002)
Mixed expiratory	SUMMA passivated stainless steel canisters	Stainless steel tube with glass beads	(Thomas et al. 1991)
Inhale (hold breath for 10 s) and forcefully expire	Glass tube	–	(Stein et al. 1996)
15 s Breath holding then exhalation	Polypropylene tubing, Gas-tight syringe	Glass trap tube—Tenax GC	(Tangerman et al. 1983)
Breath collected in bag	Tedlar bag	SPME—CAR/PDMS, DVB/PDMS, PDMS, CAR/PDMS/DVB	(Garcia et al. 2014)
Breath collected in bag	Tedlar bag Glass vial	SPME—75 µM CAR/PDMS	(Rudnicka et al. 2011)
Breath collected in bag	Smart Bag PA	NTD—Carbopack X and CMS absorbent	(Ueta et al. 2014)

## Sampling exhaled breath

During breath sampling, there is a choice made as to the portion of the breath that can be collected, and this can be broadly divided into late expiratory, end-tidal, and mixed expiratory. Mixed expiratory breath sampling encompasses the total exhaled breath which includes ‘dead space air’ (air not involved in gaseous exchange including mouth and potentially nose air) while the other breath types aim to minimise contamination from this dead space. Each of the three main types of sampling will be discussed further.

### Late expiratory breath

**Fig. 3:**
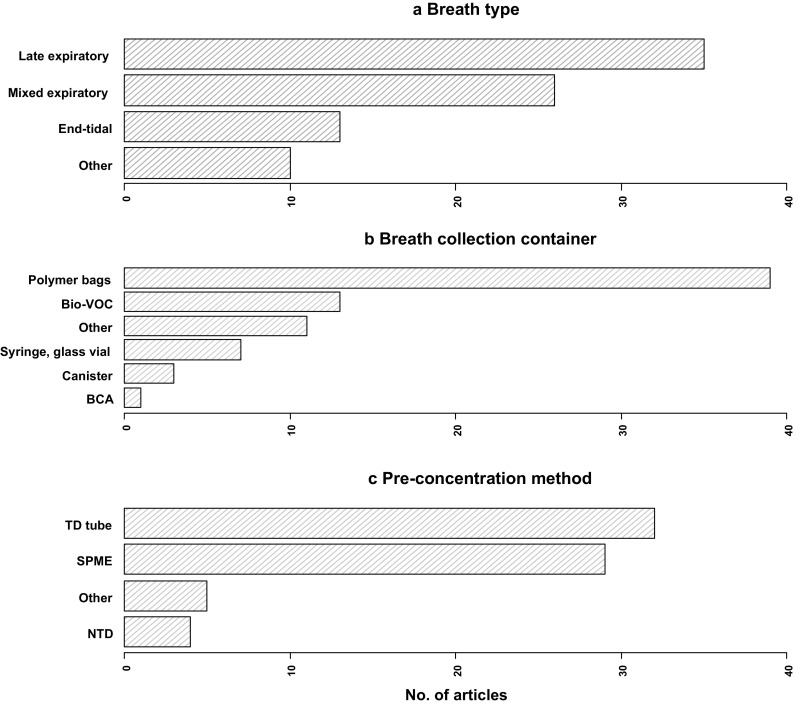
Bar charts showing the percentage distribution of **a** breath types, **b** breath collection containers, **c** pre-concentration methods reviewed

Late expiratory breath sampling involves discarding the initial portion of exhaled breath (estimated dead space) and the subsequent capture of air at the end of the breath cycle. This type of breath accounts for a large proportion of studies which is presented in Table 1 (Fig. 3a). Minimisation of dead space (Phase I in Fig. 2) sampling allows a greater relative contribution of endogenous VOCs in the resultant sample, as well as reduces the levels of exogenous VOCs. In some methods this simply mandates excluding the first few seconds of exhalation from an individual before the breath sample is collected (Castellanos et al. 2016; Sanchez and Sacks 2006). Others involve a subject breathing into a collection reservoir for the breath to flow downstream whilst the air that is close to the donor is collected and may not be as straightforward (Phillips 1997). Time-controlled breath samples have been shown to be unreliable (Miekisch et al. 2008) and also with various timings used in different studies, there is no known optimal exclusion time duration. Concerns regarding reproducibility also arise due to distinct physiological properties of individuals such as cardiac output and pulmonary ventilation which may also introduce unwanted variability even within individuals sampled repeatedly in different physiological states (Cope et al. 2004; Sukul et al. 2016). Other sources of variability which may contribute to a lack of reproducibility include breath holding and expiratory flow rate which may alter VOC concentrations (Dweik et al. 2011; Sukul et al. 2014). Thus, with several concerns associated with this type of breath, more effort is still required before it is suitable for use in the clinic. The ideal system would need to adapt to the current physiological state of each individual to collect a representative sample and minimise dead space contamination, but this would be at the cost of the simplicity and practicality of many current systems (Kwak et al. 2014; Martin et al. 2010). By contrast, the use of a pressure sensor which activates sampling during a predefined phase of expiration, as determined by individual’s expiratory pressure curve, can be used as a personalised system for sampling (Basanta et al. 2010). It is highly engineered, bulky and complex but may be a more precise alternative.

### End-tidal or ‘Alveolar’ breath

The term ‘alveolar’ should be used with caution. It is used to indicate air collected from the start to the end of phase III of the breath cycle (Fig. 2). This type of air is stated to contain high concentrations of endogenous VOCs and minimal contaminants. However, using the term ‘alveolar’ may be misleading as gas exchange models have shown that VOC exchange with the airways may be occurring (Anderson et al. 2003; Anderson and Hlastala 2007) and thus may not completely reflect alveolar concentrations. Revised terminologies for this breath type include end-tidal, end-exhaled, and end-expired (Lourenco and Turner 2014); we use end-tidal in this article. This type of breath differs from late expiratory in terms of the confidence of obtaining a representative (and personalised) end-tidal sample, using a visual cue to collect air only from phase III.

In the articles reviewed, CO_2_ visual control accounts for the most common method (see ‘Brief description’ in Table 2) used to collect end-tidal breath and it involves monitoring CO_2_ concentrations during exhalation. During phase I of exhalation (Fig. 2), CO_2_ levels are generally low but rise during transition (phase II) and subsequently approaches a plateau signalling the start of alveolar phase III. Breath CO_2_ levels can be monitored via a device known as a capnometer that enables visualisation of the various phases for guidance on when to begin breath capture. There are devices which allow manual removal of air when alveolar phase has been reached (Miekisch et al. 2010, 2008) and also automatic capture (Salvo et al. 2015). The use of CO_2_ visual control breath sampling can also be extended to ventilated patients (Schubert et al. 2005).

Since a VOC profile of mostly endogenous sample is expected, there is a considerable likelihood of detecting potentially robust markers which decreases the chances of a type I error. Similarly to late expiratory breath, airway VOCs may be lost and as not all endogenous markers originate systemically, this breath type is not suited for investigating airway diseases. A well-known endogenous (non-organic) breath marker originating from the airways is nitric oxide which has been adopted in the clinic for the characterisation of asthma (Dweik et al. 2011). Since breath collection is adapted to each individual as samples are collected at phase III, variability regarding collection of samples is minimised and thus it would be more suitable for use in a clinic due to the availability of a benchmark. There are many steps before clinic adoption, but at least it might enable comparison of data between laboratories and studies.

Rebreathing is another proposed method for achieving an end-tidal breath sample. In this approach the aim is to achieve equilibrium between a container, the airways, and bloodstream as a subject breathes. There is usually no introduction of an external source of air during this process. It is postulated that the concentration in the container reflects a concentration close to blood concentration. A comparable breath and blood ethanol concentration has been shown using this method (Jones 1983). However, this is for a single analyte and may not apply to all metabolites. A similar result was obtained by Ohlsson et al. using a variation of rebreathing known as isothermal rebreathing (Ohlsson et al. 1990). This variant aims to minimise condensation in the container and airways. A protocol for isothermal rebreathing has also been developed by (O’Hara et al. 2008). Visual representation of breath phases is absent and may not be necessary using this method as ambient air is not introduced after the first exhalation. This approach may not be suitable for clinic due to potential discomfort to patients; i.e., rising carbon dioxide levels and decreasing oxygen concentration, and would certainly not be appropriate for individuals with acute respiratory compromise (or in mechanically ventilated patients). Condensation may also impact on the stability of VOCs collected in this manner if not addressed.

### Mixed expiratory

Mixed expiratory breath can be considered as the simplest type of breath that can be obtained since it involves acquiring all phases of air exhaled as depicted in Fig. 2. It may be an attractive option due to its simplicity, however, it may not provide the best quality of breath sample due to a greater abundance of environmental, mouth, and nose contaminants. Although there are feature selection models to aid in candidate biomarker selection, unless subject numbers are very large, there may still be a considerable chance of a false positive result in this situation; i.e., identifying an exogenous VOC as a candidate marker. This has been acknowledged by several studies highlighting the need for rigorous control and reproducible sampling when obtaining breath samples (Miekisch et al. 2008; O’Hara et al. 2008; Thekedar et al. 2011).

In summary, the ideal breath sampling method would be simple, tailored to personal physiology, allow targeted selection of airway and/or alveolar air (as appropriate to the disease under study), and eliminate sampling from the dead space and environment. This combination is not yet possible, and all current methods necessitate compromise in one or more areas. As breath analysis is currently still in its infancy, and the main aim is biomarker discovery, we would propose that compromises should be minimised (and simplicity in particular sacrificed).

## Breath collection containers

Direct breath sampling onto pre-concentration materials is possible (Basanta et al. 2010), but a large proportion of the studies reviewed describe temporary storage prior to pre-concentration. The frequency of use for various storage containers is shown Fig. 3b. Polymer bags encompass the majority of breath collection containers; of which Tedlar^®^ bags (E.I. du Pont de Nemours and Company, Wilmington, DE, USA) are the most commonly used. Other polymer bags include Mylar, and aluminium bags. Bio-VOC™ Sampler (Markes International, Llantrisant, Wales, UK) (a late expiratory breath sampler), breath collection apparatus (BCA, Menssana Research Inc., Newark, NJ, USA), and glass vials (in combination with gas-tight syringes) have also been utilised for breath collection (Rattray et al. 2014). For clinical utility, the ideal collection container should be cost and user-friendly, durable, inert and importantly allow neither ingress of environmental nor egress of breath VOCs. It would also be compatible with multiple VOC trapping devices. As a case study, BCA and Bio-VOC™ Sampler devices have the same aim of collecting late expiratory air. The structure and mechanism of collection already suggests potential differences in collected compounds; i.e., BCA is an extended tubular structure where air flows downstream and the air proximal to the mouth is collected. The Bio-VOC™ Sampler is a small storage reservoir in which air is continuously displaced as exhalation proceeds. The aim of both devices is to capture breath sample that has a greater concentration of preferentially late-expiratory air. To limit condensation, the BCA has a heated component, whilst the Bio-VOC™ Sampler does not and obtained VOC profiles may vary. Other factors such as background or contaminant levels of the container should be considered in order to prevent compromising valuable human samples. Standard guidelines that take into account background contaminants may be useful. The possible variability of the breath sample during storage should also be considered and documented, as it is known that Tedlar^®^ bags absorb some constituents of breath during storage (Beauchamp et al. 2008).

## Pre-concentration methods

Pre-concentration is often necessary in order to detect VOCs that are present in breath at ppmv and lower concentrations. For some collection methods, dilution of VOCs can occur and this is particularly the case for mixed expiratory breath samples. Commonly used pre-concentration methods include sorbent-containing thermal desorption (TD) tubes, the employment of solid phase microextraction (SPME), as well as needle trap devices (NTDs) and these are discussed below.

### Thermal desorption (TD) tubes

TD tubes are popular for pre-concentrating VOCs and account for almost half of the pre-concentration methods published to date (Fig. 3c). Sorbents can be manually packed into the tubes or tubes can be purchased pre-packed from suppliers. Commonly used sorbents include Tenax TA & GR, Carbograph 5TD, Carboxen. Due to the distinct properties of these materials there is important variability in the range of volatiles that can be trapped, as well as the stability of sorbent-compound interaction. Sample volume should be considered in order to prevent breakthrough and subsequent loss of analytes. Strong sorbents such as Carboxen are suitable for trapping very volatile organic compounds (~C_2_–C_4_) while Tenax sorbents trap less volatile VOCs in breath (~C_7_–C_15_) (Dettmer and Engewald 2002). Some factors that need to be considered include whether to use single or multi-bed sorbents which will depend on analytes of interest, and also the quantity of sorbent used. Whilst multi-bed sorbents can trap a larger range of different chemical species, both in terms of volatility but also polarity, analyte-sorbent interactions at the interfaces of the packed beds can affect reproducibility and thus compound recovery, and perhaps stability during storage (Kang and Paul Thomas 2016).

When breath has been temporarily stored in for example polymer bags, VOC-capture proceeds by attaching one end of the TD tube to the bag and the other end to a pump which functions to ‘pull breath’ from the bag across the sorbent. Whilst the use of these sorbent-containing TD tubes is stated to be highly sensitive, it can be quite time consuming. Also, sorbents like Carboxen are hydrophilic thus retain moisture which can negatively affect the quantitative capture of some analytes. Introducing a dry-purging step especially when using a hydrophilic sorbent may be a solution (Gawlowski et al. 2000). Concerns such as the stability of VOCs trapped onto sorbent materials, storage and also logistics are apparent. It has been shown that these tubes can be stored for up to 2 weeks prior to analysis (Harshman et al. 2016; van der Schee et al. 2013) whilst another study suggests longer (Kang and Paul Thomas 2016). Samples collected using this method can be analysed in the laboratory present in hospitals provided that the required analytical platform is available which would require a significant investment. Alternatively, tubes can be temporarily stored and sent to laboratories off-site. Either way, this is readily adaptable for use in the clinic.

### Solid phase microextraction (SPME)

SPME is a pre-concentration technique developed by Pawliszyn et al. which has found application for use in breath analysis (Grote and Pawliszyn 1997). It involves exposing a coated fused-silica fibre to the headspace of samples. Coating materials include amongst others polydimethylsiloxane (PDMS) and polyacrylate (PA). These coatings trap VOCs by absorption/adsorption mechanisms (Vas and Vekey 2004). Sampling is usually completed when equilibrium is established between the fibre and the sample which is stated to be fast in the case of volatile analytes. The time to equilibrium depends on several factors including fibre type, thickness, length, and agitation. Common combinations of SPME coatings, as tabulated in the pre-concentration summaries (Tables 1, 2, 3), include 75 µm/85 µm CAR/PDMS for gases and low molecular weight compounds (MW 30–225), 100 µm PDMS for volatiles (MW 60–275), 65 µm PDMS/DVB for volatiles, amines, & nitro-aromatic compounds (MW 50–300), 85 µm PA for polar semi-volatiles (MW 80–300), 7 µm PDMS for non-polar high molecular weight compounds (MW 125–600), 30 µm PDMS for non-polar semi-volatiles, and 60 µm CW for alcohols and polar compounds (MW 40–275) (Sigma-Aldrich, Gillingham, UK). This method has been stated to have a comparable sensitivity to TD tubes but may be limited by the amount of coating and thickness of fibres (Vas and Vekey 2004).

### Needle trap devices (NTDs)

NTDs are also utilised for the capture of analytes from exhaled breath. Briefly, sorbent materials are confined within a needle-like device and breath is ‘pulled’ through the needle to capture VOCs. The method aims to encompass the best features from SPME and TD tubes i.e. reduced sampling times while retaining adequate sensitivity (Lord et al. 2010). This method is discussed in detail elsewhere (Filipiak et al. 2012; Trefz et al. 2012, 2013). Similarly to TD tubes, sensitivity can be improved by increasing sampling volume (Trefz et al. 2013). Samples obtained using both SPME (Chai and Pawliszyn 1995; Grote and Pawliszyn 1997) and NTDs (Mieth et al. 2009) are reported to be stable for a couple of hours before significant losses are observed.

## Recommendations and future directions

We have summarised the analytical pipelines that are commonly utilised for the off-line capture and pre-concentration of breath for VOC analysis. It is clear from this review that prior to conducting a breathomics study, the important sampling considerations include selection of the relevant breath fraction, the type of breath collecting container (if used), and pre-concentration technique.

From this literature survey we have seen that the methods of collecting late expiratory breath differs between studies and this may be due to an actual lack of proper definition and thus may be a contributing factor to the heterogeneous results which are reported (Dent et al. 2013; Phillips et al. 2003; Poli et al. 2005). Some studies associate the term ‘alveolar’ with their breath samples, and although in these studies the first few volumes of captured breath are discarded, this should be considered as late expiratory breath. We consider that breath samples collected under control, such as the CO_2_ monitoring method shown in Fig. 2 (i.e., from the start to end of phase III), may be described as ‘alveolar’ or end-tidal as the collection of the breath is bespoke to the person under analysis. This is not to suggest that late expiratory breath is not useful. On the contrary, as it does not use such complex CO_2_ monitoring, it does have the advantages such as ease of collection and it is also inexpensive and these deem it an attractive option. The aforementioned varied late expiratory definitions include different timings for exclusion of dead space air and the use of breath sampling manoeuvres such as breath holding and forced expirations which are stated to influence VOC content (Bikov et al. 2014; Dweik et al. 2011; Larstad et al. 2007; Thekedar et al. 2011) (see ‘Brief description’ in Table 1). This lack of uniformity most likely affects the reproducibility of acquired results. The goal of analysing the late expiratory breath is to capture a sample with a greater endogenous contribution. It would be more useful to obtain this type of sample with confidence using the controls that are available such as capnography or a set-defined late expiratory breath protocol. This is because this is specific to the individual, as each person will have a different breath profile due to size of their lungs, fitness and medication/disease.

Consistency with breath collection containers will also be important. A device having a heating component to prevent or minimise condensation effect and another without this property may culminate in the reporting of different results. Therefore adequate development and handling of these devices is required to maintain quality. Minimisation of condensation, removal of background contaminants and optimisation of storage time should be prioritised before use for studies. (Beauchamp et al. 2008); (Phillips 1997; Phillips et al. 2003). This should also be extended to pre-concentration techniques.

Herbig and Beauchamp suggested a framework for standardisation of reporting for breath sampling methods (Herbig and Beauchamp 2014), whereby the *breath type* and *method of obtaining a breath type* are reported to enable comparison between studies. That such standardisation is possible is showcased by the HbA1c test for determining glucose levels in blood. Here also, similar reproducibility difficulties were experienced as a result of varying results across laboratories. This resulted in primary reference laboratories being established to work together nationally and then globally to deliver standardisation of this test (John et al. 2007). Only with cooperation between laboratories can the differences in breath type definition be fully explored and defined. Moreover, direct comparison of collection practices on a global scale will be required to establish a consensus on important parameters.

The need for breath sampling standardisation is stimulating engagement activity within the community as shown by a recent survey (Beauchamp 2015). Recently, a breath sampler was developed with input from a broad consortium of breath researchers and engineers which may aid in contributing to the sought after homogeneity in sampling within the breathomics community (http://www.breathe-free.org). Finally, it is important to acknowledge that the collection of a certain type of breath should be linked to the clinical question under investigation.

## Summary and conclusion

We have reviewed 110 articles describing various breath sampling methodologies and assessed them in terms of breath type collected, the containers used for collecting breath, and pre-concentration methods employed. It is clear from this research that the breath community need to converge in order to make improvements along the various steps of the breathomics analysis pipeline (as depicted in Fig. 1). We found that late expiratory breath is the most common breath type and is typically obtained based on crude estimations. These estimations of late expiratory breath are different in distinct studies and we suggest that standardisation of analysis protocols should be considered when acquiring this breath type. Breath sampling with the use of some form of guidance to provide confidence would certainly aid in improving the quality of the obtained sample. Focus on the clinical question may also help in determining the best type of breath sample to be collected.

We believe that improvement in quality along the various steps in the pipeline will aid in realising the translational potential of breath research from the laboratory and into the clinic.

## Electronic supplementary material

Below is the link to the electronic supplementary material.

Supplementary material 1 (DOCX 69 KB)

## Abbreviations

BCA: Breath collection apparatus
CAR: Carboxen
CW: Carbowax
DVB: Divinylbenzene
DMS: Differential mobility spectrometry
ECD: Electron capture detector
FID: Flame ionisation detector
GC-MS: Gas chromatography-mass spectrometry
MW: Molecular weight
NTDs: Needle trap devices
PA: Polyacrylate
PDMS: Polydimethylsiloxane
ppmv: Parts per million volume
ppbv: Parts per billion volume
SPME: Solid phase microextraction
TD: Thermal desorption
Tof: Time of flight
VOCs: Volatile organic compounds
