# Supplementary material for: Exhaled breath analysis: a review of ‘breath-taking’ methods for off-line analysis
Source: Metabolomics. 2017 Aug 19;13(10):110. doi: 10.1007/s11306-017-1241-8 (PMC5563344; doi:10.1007/s11306-017-1241-8)
Supplement: Supplementary file 1 — Supplementary material 1 (DOCX 69 KB) [file 11306_2017_1241_MOESM1_ESM.docx]

**Exhaled breath analysis: a review of ‘breath-taking’ methods for off-line analysis**

Oluwasola Lawal^1,2^, Waqar M. Ahmed^1,2^, Tamara M.E. Nijsen^2^, Royston Goodacre^3^, Stephen J. Fowler^1,4^

Author affiliations

^1^ Division of Infection, Immunity and Respiratory Medicine, School of Biological Sciences, Faculty of Biology, Medicine and Health, The University of Manchester, Manchester, United Kingdom

^2^Philips Research, Royal Philips B.V., Eindhoven, The Netherlands

^3^School of Chemistry, Manchester Institute of Biotechnology, The University of Manchester, Manchester, United Kingdom

^4^ Manchester Academic Health Science Centre, The University of Manchester and University Hospital of South Manchester NHS Foundation Trust, Manchester, United Kingdom

Correspondence: stephen.fowler@manchester.ac.uk

Supplementary material

**Selection process of included manuscripts**

**
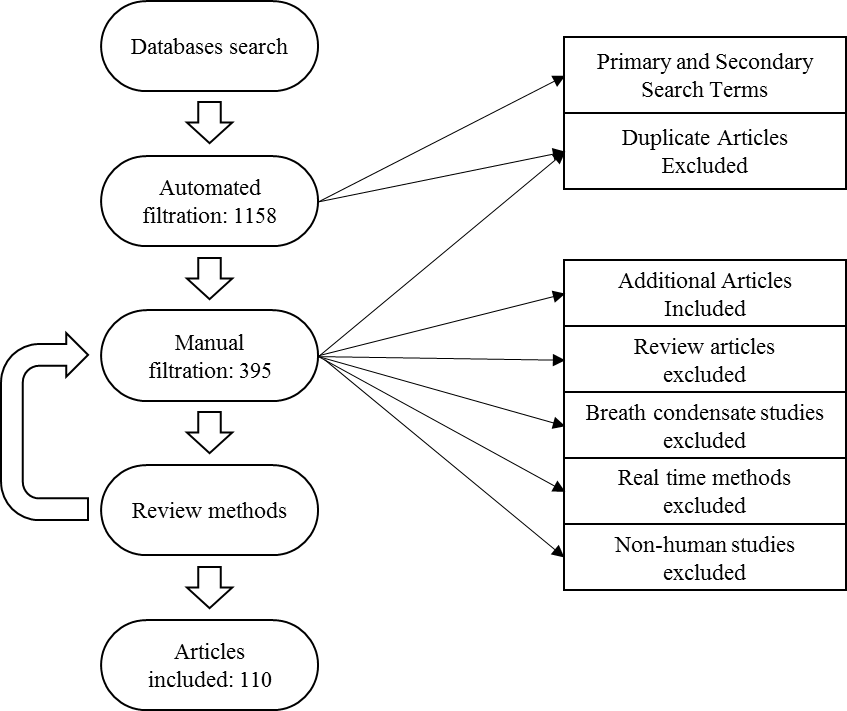
**

**Figure S1.** *Flow diagram highlighting the method used to compile a collection of relevant bibliography*

**Table S1: Studies that collected other breath types**

| **Brief description** | **Breath collection container** | **Pre-concentration method** | **Reference** |
| --- | --- | --- | --- |
| *Air from ipsilateral and contralateral lungs* | Gas-tight syringe  Airtight vial | SPME - 75µM CAR/PDMS | (Wang et al., 2014) |
| *Distal airways air* | Face mask | TD tube - Tenax TA/Carbograph 1TD | (Turner et al., 2013) |
| *Bronchoscopic air sampling* | Tedlar bag | SPME – fiber not specified | (Santonico et al., 2012) |
| *Peripheral airways and Alveoli* | Stainless steel SUMMA canister | - | (Delfino et al., 2003) |
| **Distal intratracheal air* | - | TD tube - Tenax TA/Carbotrap | (Fowler et al., 2015) |
| **Air from ventilator circuit* | Tedlar | TD tube – Carbograph 1TD/Carbopack X | (Schnabel et al., 2015) |
| **Air from ventilator circuit* | - | TD tube - Tenax TA | (Gao et al., 2016) |
| **Air from ventilator circuit* | - | TD tube - Tenax GR | (Bos et al., 2014) |
| **Air from ventilator circuit* | - | Ceramic trap – activated charcoal | (Schubert et al., 1998) |
| **Air from ventilator circuit* | - | TD tube – Tenax GR,  Tenax GR/Carbograph 5TD | (van Oort et al., 2017) |

**References**

Bos, L.D., Wang, Y., Weda, H., Nijsen, T.M., Janssen, A.P., Knobel, H.H., Vink, T.J., Schultz, M.J., and Sterk, P.J. (2014). A simple breath sampling method in intubated and mechanically ventilated critically ill patients. Respir Physiol Neurobiol *191*, 67-74.

Delfino, R.J., Gong, H., Linn, W.S., Hu, Y., and Pellizzari, E.D. (2003). Respiratory symptoms and peak expiratory flow in children with asthma in relation to volatile organic compounds in exhaled breath and ambient air. J Expo Anal Environ Epidemiol *13*, 348-363.

Fowler, S.J., Basanta-Sanchez, M., Xu, Y., Goodacre, R., and Dark, P.M. (2015). Surveillance for lower airway pathogens in mechanically ventilated patients by metabolomic analysis of exhaled breath: a case-control study. Thorax *70*, 320-325.

Gao, J., Zou, Y., Wang, Y., Wang, F., Lang, L., Wang, P., Zhou, Y., and Ying, K. (2016). Breath analysis for noninvasively differentiating Acinetobacter baumannii ventilator-associated pneumonia from its respiratory tract colonization of ventilated patients. J Breath Res *10*, 027102.

Santonico, M., Lucantoni, G., Pennazza, G., Capuano, R., Galluccio, G., Roscioni, C., La Delfa, G., Consoli, D., Martinelli, E., Paolesse, R.*, et al.* (2012). In situ detection Of lung cancer volatile fingerprints using bronchoscopic air-sampling. Lung Cancer *77*, 46-50.

Schnabel, R., Fijten, R., Smolinska, A., Dallinga, J., Boumans, M.L., Stobberingh, E., Boots, A., Roekaerts, P., Bergmans, D., and van Schooten, F.J. (2015). Analysis of volatile organic compounds in exhaled breath to diagnose ventilator-associated pneumonia. Sci Rep *5*, 17179.

Schubert, J.K., Muller, W.P., Benzing, A., and Geiger, K. (1998). Application of a new method for analysis of exhaled gas in critically ill patients. Intensive Care Med *24*, 415-421.

Turner, M.A., Bandelow, S., Edwards, L., Patel, P., Martin, H.J., Wilson, I.D., and Thomas, C.L. (2013). The effect of a paced auditory serial addition test (PASAT) intervention on the profile of volatile organic compounds in human breath: a pilot study. J Breath Res *7*, 017102.

van Oort, P.M., Nijsen, T., Weda, H., Knobel, H., Dark, P., Felton, T., Rattray, N.J., Lawal, O., Ahmed, W., Portsmouth, C.*, et al.* (2017). BreathDx - molecular analysis of exhaled breath as a diagnostic test for ventilator-associated pneumonia: protocol for a European multicentre observational study. BMC Pulm Med *17*, 1.

Wang, C.S., Dong, R., Wang, X.Y., Lian, A.L., Chi, C.J., Ke, C.F., Guo, L., Liu, S.S., Zhao, W., Xu, G.W.*, et al.* (2014). Exhaled volatile organic compounds as lung cancer biomarkers during one-lung ventilation. Scientific Reports *4*.
